# Sodium fluoride causes oxidative stress and apoptosis in the mouse liver

**DOI:** 10.18632/aging.101257

**Published:** 2017-06-28

**Authors:** Yujiao Lu, Qin Luo, Hengmin Cui, Huidan Deng, Ping Kuang, Huan Liu, Jing Fang, Zhicai Zuo, Junliang Deng, Yinglun Li, Xun Wang, Ling Zhao

**Affiliations:** ^1^ College of Veterinary Medicine, Sichuan Agricultural University, Wenjiang, Chengdu 611130, China; ^2^ Key Laboratory of Animal Diseases and Environmental Hazards of Sichuan Province, Sichuan Agriculture University, Wenjiang, Chengdu 611130, China

**Keywords:** sodium fluoride, oxidative stress, apoptosis, TNF-R1 signaling pathway, liver, mouse

## Abstract

The current study was conducted to investigate the effect of sodium fluoride (NaF) on the oxidative stress and apoptosis as well as their relationship in the mouse liver by using methods of flow cytometry, quantitative real-time polymerase chain reaction (qRT-PCR), western blot, biochemistry and experimental pathology. 240 four-week-old ICR mice were randomly divided into 4 groups and exposed to different concentration of NaF (0 mg/kg, 12 mg/kg, 24 mg/kg and 48 mg/kg) for a period of 42 days. The results showed that NaF caused oxidative stress and apoptosis. NaF-caused oxidative stress was accompanied by increasing reactive oxygen species (ROS) and malondialdehyde (MDA) levels, and decreasing mRNA expression levels and activities of superoxide dismutase (SOD), catalase (CAT), glutathione (GSH), glutathione peroxidase (GSH-PX) and glutathione-s-transferase (GST). NaF induced apoptosis via tumor necrosis factor recpter-1 (TNF-R1) signaling pathway, which was characterized by significantly increasing mRNA and protein expression levels of TNF-R1, Fas associated death domain (FADD), TNFR-associated death domain (TRADD), cysteine aspartate specific protease-8 (caspase-8) and cysteine aspartate specific protease-3 (caspase-3) in dose- and time-dependent manner. Oxidative stress is involved in the process of apoptotic occurrence, and can be triggered by promoting ROS production and reducing antioxidant function. NaF-caused oxidative stress and apoptosis finally impaired hepatic function, which was strongly supported by the histopathological lesions and increased serum alanine amino transferase (ALT), aspartic acid transferase (AST), alkaline phosphatase (AKP) activities and TBIL contents.

## INTRODUCTION

Fluoride distributes very extensively in the natural environment, and is widely used among industry, agriculture as well as medicine [1]. Fluorine is one of the essential trace elements for human body [2]. Moderate levels of fluorine or fluoride ingestion can decrease the incidence of dental caries and promote the development of bones, but there are a number of adverse effects on human health with fluorine or fluoride chronic ingestion at high doses [3]. The main pathway of fluoride poisoning is through drinking water, and high concentration fluoride is often associated with soft, alkaline, and calcium-deficient waters [4]. Other pathways of fluorine- or fluoride-entered body include food, industrial exposure, drugs, cosmetics, etc [5]. Fluoride toxicity targets to not only bone and teeth, but also soft tissues [6]. Previous studies have proved that fluorine can induce genotoxicity, cytotoxicity, immunotoxicity, oxidative damage, apoptosis and lesions in the broiler peripheral blood [7–10], liver [11, 12], kidney [13–15], thymus [16], spleen [17–19], bursa of Fabricius [20], cecal tonsil [21–25], and intestine [26–30], and in the mouse spleen and kidney [31–35].

Reactive oxygen species (ROS), as a byproduct of the metabolic process, can be scavenged by many antioxidative defense components under normal condition [36]. Imbalance between ROS and antioxidants is referred to as oxidative stress [37]. Fluoride is known to be an inhibitor of the antioxidant enzymes, which in turn promote the accumulation of ROS [38, 39]. NaF-altered ROS production levels and antioxidative parameters have been systematically observed in the mouse kidney [35]. Reports have indicated that fluoride exposure can induce oxidative stress in liver [12, 40, 41], kidney [35, 41], testicle [42], spleen [17], brain [43], heart [44] and cecal tonsil [25], and reduce the activities of superoxide dismutase (SOD), catalase (CAT), glutathione peroxidase (GSH-PX) and glutathione-s-transferase (GST) in the liver of broiler, fish, rabbit and rat [12, 40, 41, 45]. Zhou et al. has also found that fluoride can induce oxidative stress in the liver of female mice after 70 days of fluoride treatment [46]. Although there are reports on the relationship between fluoride and oxidative stress, very limited systematic studies are focused on the molecular mechanism of NaF-induced hepatic oxidative stress in mice.

It is well known that the ROS and oxidative stress can work as the inducer of apoptosis [47–49]. Apoptosis is an essential physiological process that plays a critical role in regulating growth and immune response via gene and/or protein expression [32]. Fluorine- or fluoride-induced apoptosis has been reported in vivo [11, 16, 20, 24, 33, 47–49]. Researches in liver have showed that fluoride increases caspase-3, caspase-8, caspase-9 and bax protein expression levels, and reduces the bcl-2 protein expression [50–52]. Death receptor pathway is one of the main apoptosis signal pathways [53], which belongs to extrinsic apoptosis pathway. Extrinsic apoptosis can be initiated by the binding of lethal ligands to various death receptors. For example, FasL binds to Fas, TNFα-related apoptosis-inducing ligand (TRAIL) can binds to TRAIL recptor-1/2 (TRAIL-R1/2), and TNF-α can interact with TNF-R1 and TNF-R2 [54]. Recent studies have indicated that fluoride can induce apoptosis in the liver, but there are no reports on the relationship between fluoride-induced hepatocyte apoptosis and death receptor pathway at present.

Based on the above-mentioned references, very limited systematic reports are focused on the relationship between fluoride-induced oxidative stress and apoptosis in the mouse liver. Therefore, this study was conducted to observe the possible mechanism of NaF-induced oxidative stress and apoptosis as well as the relationship between NaF-induced oxidative damage and apoptosis by detecting ROS levels and MDA contents, and mRNA expression levels and activities of SOD, CAT, GSH, GSH-PX, GST as well as the mRNA and protein expression levels of tumor necrosis factor recpter-1 (TNF-R1), tumor necrosis factor recpter-2 (TNF-R2), Fas associated death domain (FADD), TNFR-associated death domain (TRADD), caspase-8 and caspase-3 using methods of flow cytometry, quantitative real-time polymerase chain reaction (qRT-PCR), western blot, biochemistry and experimental pathology.

## RESULTS

### Histopathological lesions in the liver

**Figure 1:**
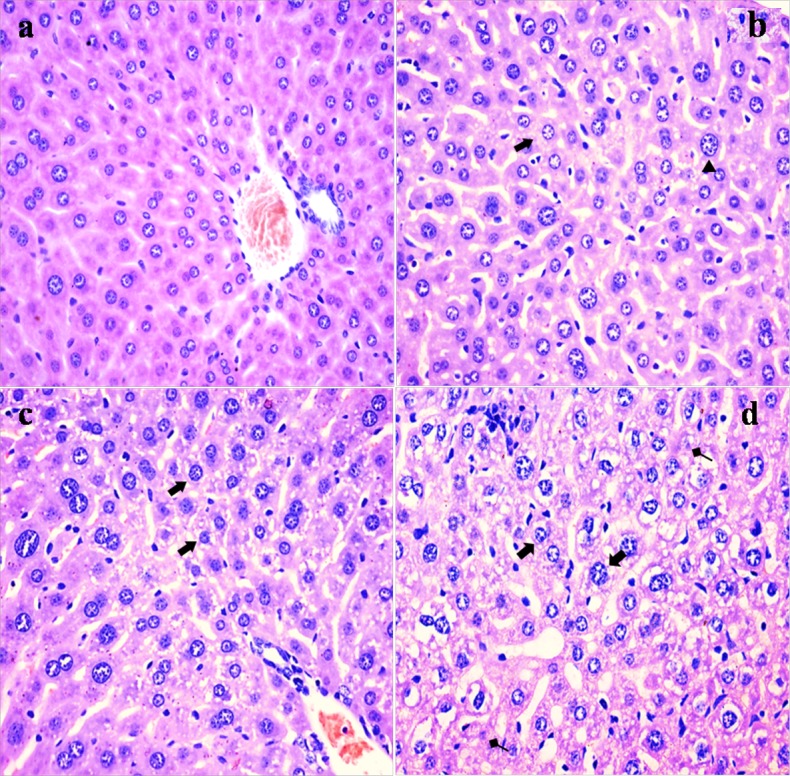
Histopathological changes in the liver at 21 days of the experiment (**a**) The control group (H&E × 400). (**b**) The 12 mg/kg group. Hepatocytes are swelled (▲) and show slight granular and vacuolar degeneration (⇑, H&E ×400). (**c**) The 24 mg/kg group. Hepatocytes show granular and vacuolar degeneration (⇑, H&E × 400). (**d**) The 48 mg/kg group. Hepatocytes show obvious granular and vacuolar degeneration (⇑). Necrotic hepatic cells (↑) are observed (H&E × 400).

**Figure 2:**
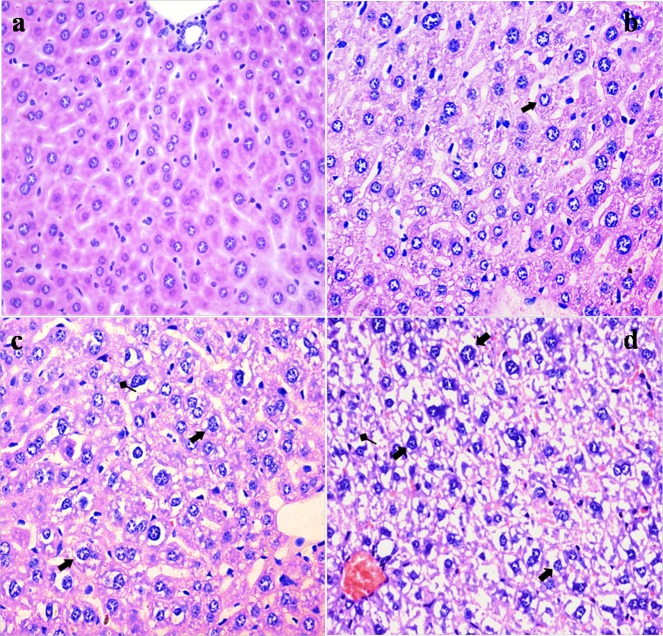
Histopathological changes in the liver at 42 days of the experiment (**a**) The control group (H&E × 400). (**b**) The 12 mg/kg group. Hepatocytes show granular and vacuolar degeneration (⇑, H&E × 400). (**c**) The 24 mg/kg group. Hepatocytes show obvious granular and vacuolar degeneration (⇑). Also, necrotic hepatocytes are observed (↑, H&E × 400). (**d**) The 48 mg/kg group. Hepatocytes show marked vacuolar degeneration (⇑). Necrotic hepatocytes are observed (↑). And hepatic cords are disorganized or disappeared (H&E × 400).

NaF resulted in histopathological lesions in a dose- and time-dependent manner. Lesions included hepatocellular granular degeneration, vacuolar degeneration and necrosis. In the granular and vacuolar degenerated hepatocytes, tiny particles and small or large vacuoles were appeared in the cytoplasm (Figure 1). Karyorrhexis, karyolysis and hypochromatosis were appeared in the necrotic hepatocytes (Figure 2). The above lesions were not observed in the control group.

### Changes of hepatic functional parameters

**Figure 3:**
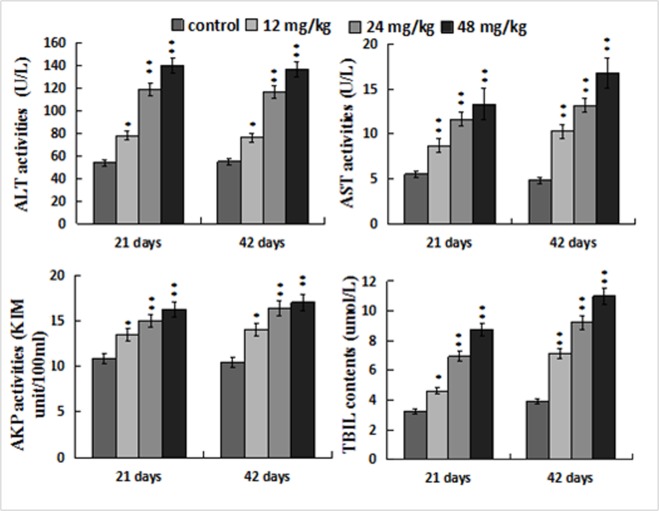
Changes of the serum ALT, AST, AKP activities and TBIL contents Data are presented with the mean ± standard deviation (n=8). *p < 0.05, compared with the control group; **p < 0.01, compared with the control group.

Figure 3 showed that ALT, AST and AKP activities, and TBIL contents were increased (*p* < 0.05 or *p* <; 0.01) in the 12, 24, 48 mg/kg groups when compared with those in the control group at 21 and 42 days of the experiment.

### Changes of oxidative damage parameters in the liver

**Figure 4:**
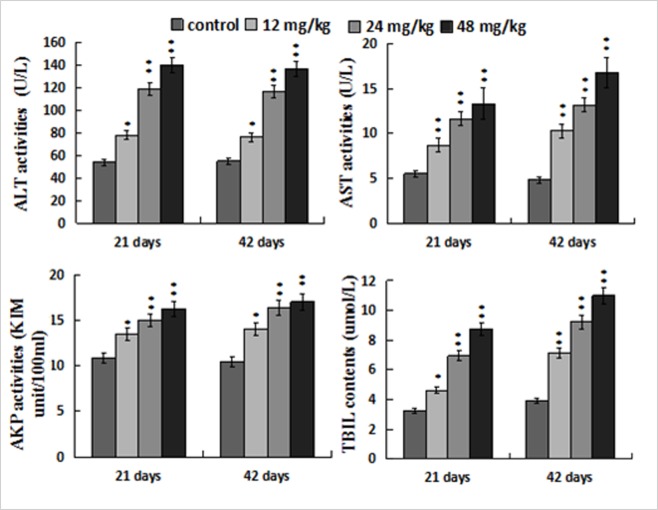
Changes of oxidative damage experiment parameters in the liver Data are presented with the mean ± standard deviation (n=8). *p < 0.05, compared with the control group; **p < 0.01, compared with the control group.

The MDA contents were significantly elevated (*p* < 0.05 or *p* < 0.01) in the 12, 24, 48 mg/kg groups in comparison with those in the control group at 21 and 42 days of the experiment. The SOD and GSH-PX activities were significantly lower (*p* < 0.05 or *p* < 0.01) in the 24, 48 mg/kg groups at 21 days of the experiment and in the 12, 24, 48 mg/kg groups at 42 days of the experiment than those in the control group. The GSH contents and the GST activities were decreased (*p* < 0.05 or *p* < 0.01) in the three NaF-treated groups at 21 and 42 days of the experiment. The CAT activities were obviously declined (*p* < 0.05) only in the 48mg/kg group at 42 days of the experiment. The results were shown in Figure 4.

### Changes of antioxidant enzyme mRNA expression levels in the liver

**Figure 5:**
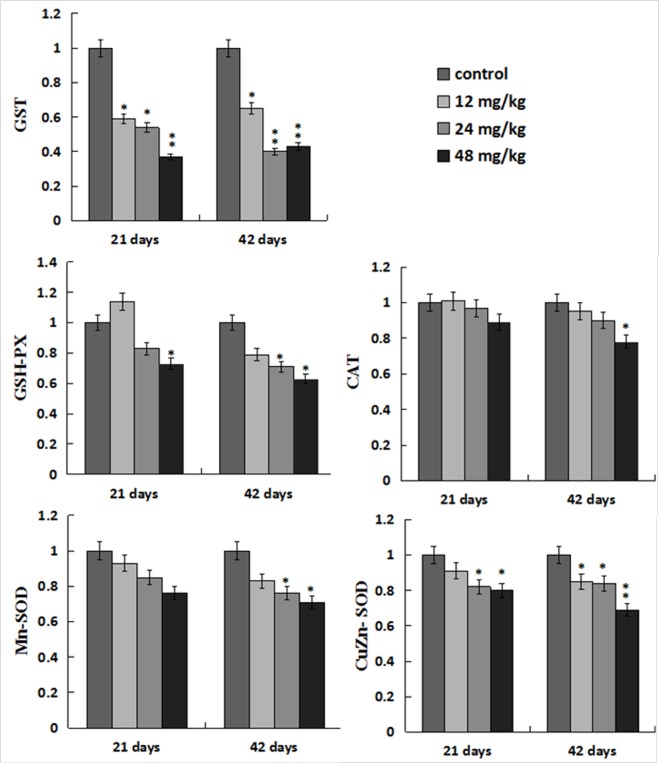
Changes of antioxidant enzymes mRNA expression levels in the liver Data are presented with the mean ± standard deviation (n=8). *p < 0.05, compared with the control group; **p < 0.01, compared with the control group.

The GST mRNA expression levels were decreased (*p* < 0.05 or *p* < 0.01) in the three NaF-treated groups at 21 and 42 days of the experiment. The GSH-Px mRNA expression levels were lower (*p* < 0.05) in the 48mg/kg group at 21 days of the experiment and in the 24, 48mg/kg groups at 42 days of the experiment than those in the control group. The Mn-SOD mRNA expression levels were decreased in the 24, 48mg/kg groups at 42 days of the experiment, and the CuZn-SOD mRNA expression was decreased (*p* < 0.05 or *p* < 0.01) in the 12, 24, 48 mg/kg groups at 21 and 42 days of the experiment except in the 12mg/kg group at 21 days of the experiment when compared with those in the control group. The CAT mRNA expression levels were lower (*p* < 0.05) only in the 48 mg/kg group than those in the control group at 42 days of the experiment. The results were shown in Figure 5.

### Changes of ROS production levels in the liver

**Figure 6:**
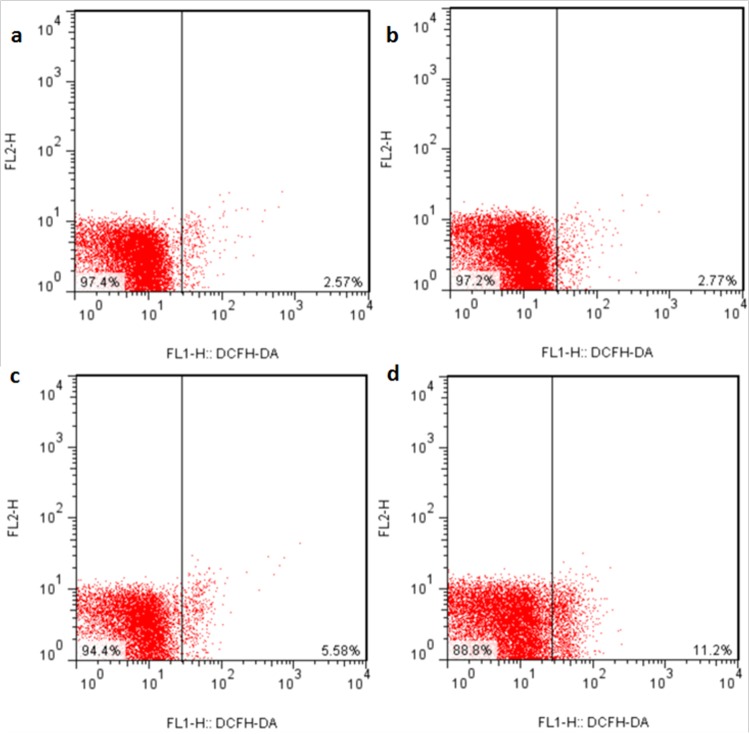
ROS production levels in the liver at 21 days of the experiment Control group (**a**), 12mg/kg (**b**), 24mg/kg (**c**), 48mg/kg (**d**).

**Figure 7:**
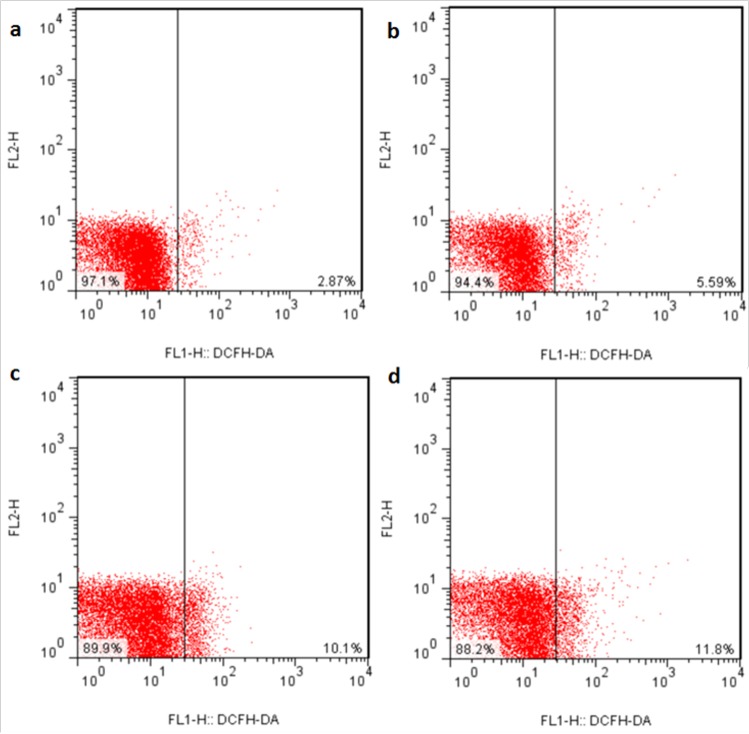
ROS production levels in the liver at 42 days of the experiment Control group (**a**), 12mg/kg (**b**), 24mg/kg (**c**), 48mg/kg (**d**).

**Figure 8:**
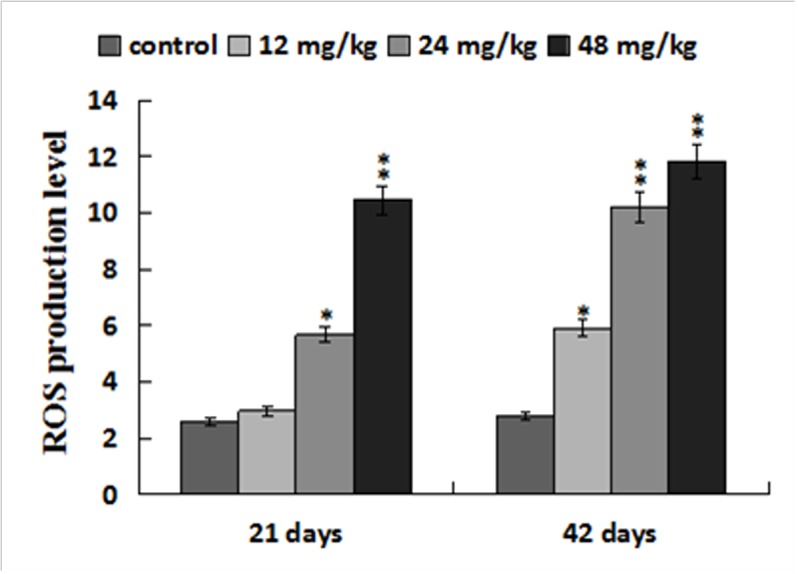
ROS production levels in the liver Data are presented with the mean ± standard deviation (n=8). *p < 0.05, compared with the control group; **p < 0.01, compared with the control group.

The ROS production levels were significantly increased (*p* < 0.05 or *p* < 0.01) in the three NaF-treated groups at 21 and 42 days of the experiment except in the 12 mg/kg group at 21days of the experiment when compared with those in the control group. The results were shown in Figures 6, 7 and 8.

### Changes of apoptosis percentages in the liver

**Figure 9:**
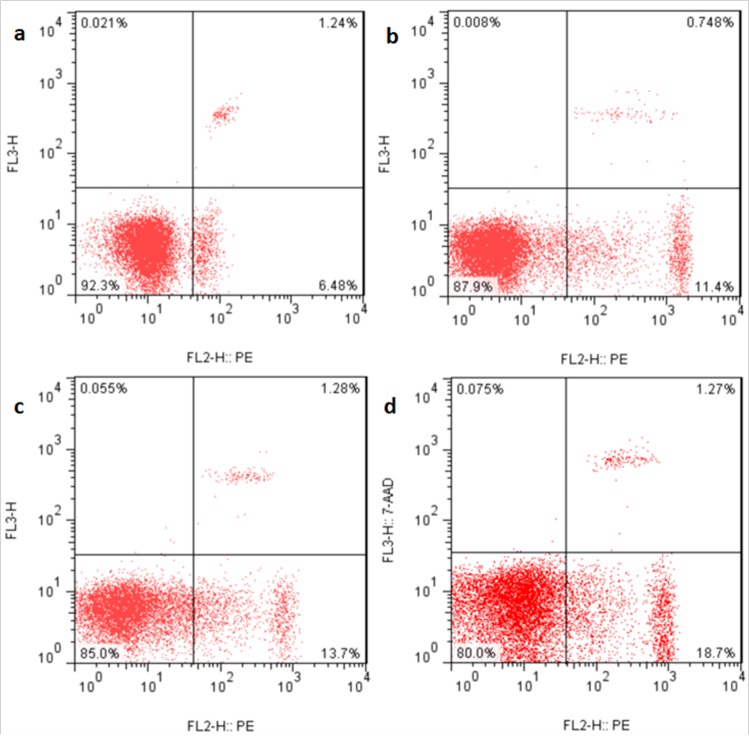
Apoptosis in the liver at 21 days of the experiment Control group (**a**), 12mg/kg (**b**), 24mg/kg (**c**), 48mg/kg (**d**).

**Figure 10:**
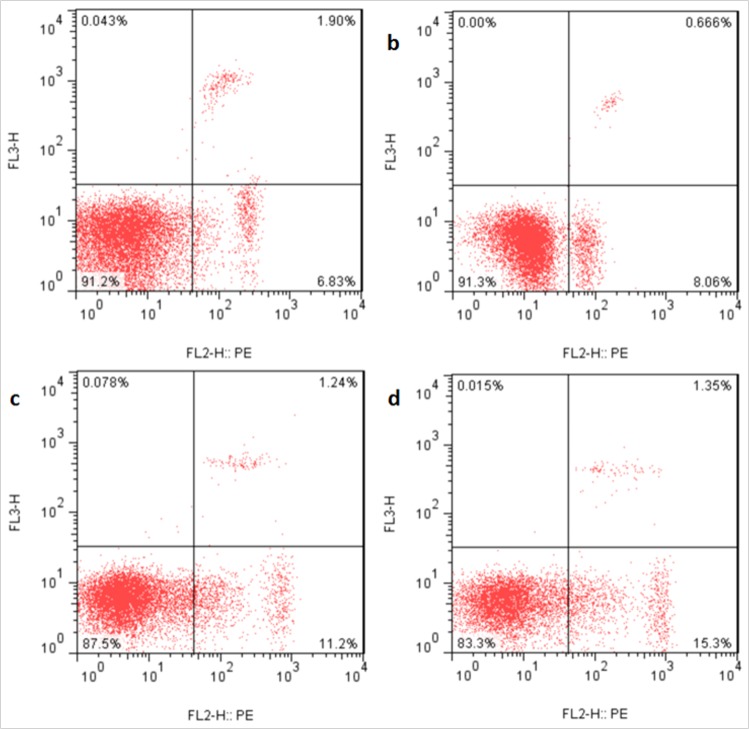
Apoptosis in the liver at 42 days of the experiment Control group (**a**), 12mg/kg (**b**), 24mg/kg (**c**), 48mg/kg (**d**).

**Figure 11:**
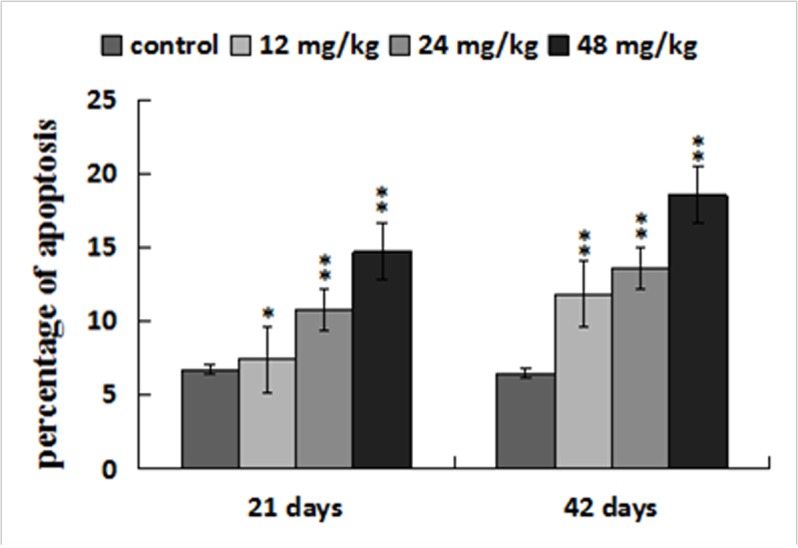
Percentage of apoptosis in the liver Data are presented with the mean ± standard deviation (n=8). *p < 0.05, compared with the control group; **p < 0.01, compared with the control group.

The apoptotic percentages were significantly increased (*p* < 0.05 or *p* < 0.01) in the three NaF-treated groups at 21 and 42 days of the experiment in comparison with those in the control group. The results were shown in Figures 9, 10 and 11.

### Changes of mRNA expression levels of parameters associated to death receptor pathway in the liver

**Figure 12:**
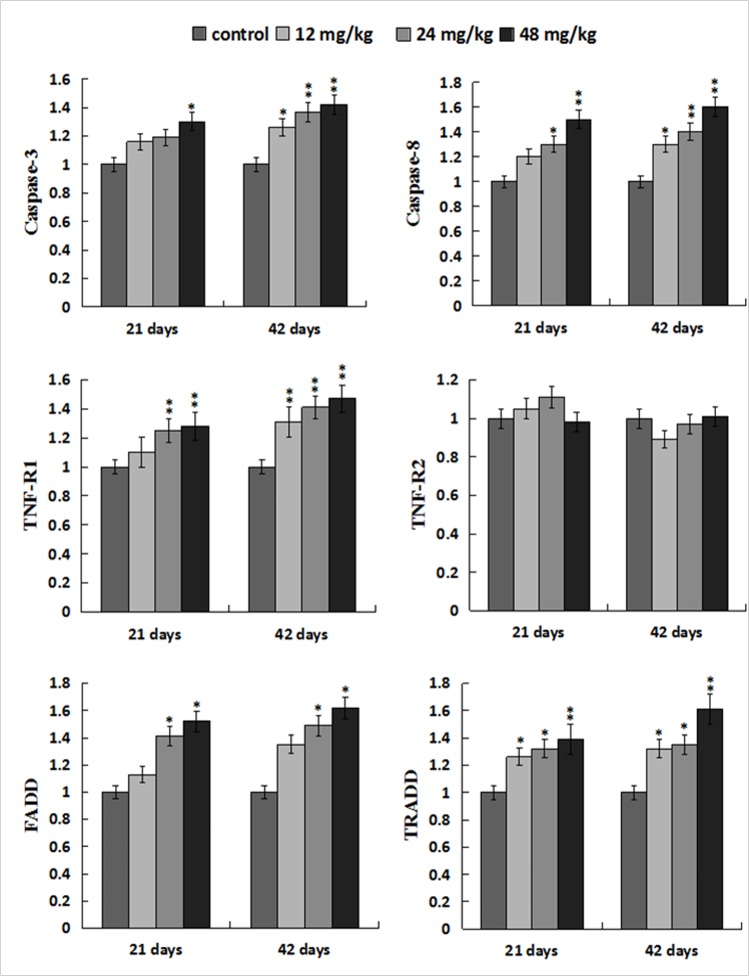
Changes of mRNA expression levels of apoptotic parameters associated to TNF-α signaling pathway in the liver Data are presented with the mean ± standard deviation (n=8). *p < 0.05, compared with the control group; **p < 0.01, compared with the control group.

The caspase-3 mRNA expression levels were higher (*p* < 0.05) in the 48mg/kg group at 21 days of the experiment and were significantly higher (*p* < 0.05 or *p* < 0.01) in the 12, 24, 48 mg/kg groups at 42 days of the experiment than those in the control group. The caspase-8 and TNF-R1 mRNA expression levels were increased (*p* < 0.05 or *p* < 0.01) in the 12, 24, 48 mg/kg groups at 21and 42 days of the experiment except in the 12 mg/kg group at 21 days of the experiment. However, the TNF-R2 mRNA expression levels were not obviously changed when compared with those in the control group. The FADD mRNA expression levels were elevated (*p* < 0.05) in the 24, 48mg/kg groups at 21 and 42 days of the experiment. The TRADD mRNA expression levels were higher (*p* < 0.05 or *p* < 0.01) in the 12, 24, 48 mg/kg groups at 21 and 42 days of the experiment than those in the control group. The results were shown in Figure 12.

### Changes of protein expression levels of parameters associated to death receptor pathway in the liver

**Figure 13:**
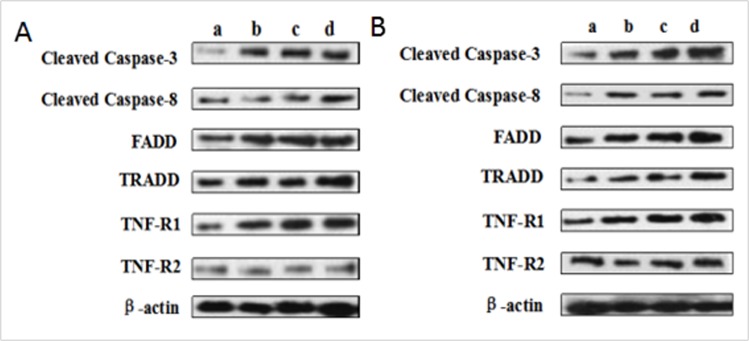
(**A**) The western blot assay at 21 days of the experiment (**B**) The western blot assay at 42 days of the experiment. Control group (**a**), 12mg/kg (**b**), 24mg/kg (**c**), 48mg/kg (**d**).

**Figure 14:**
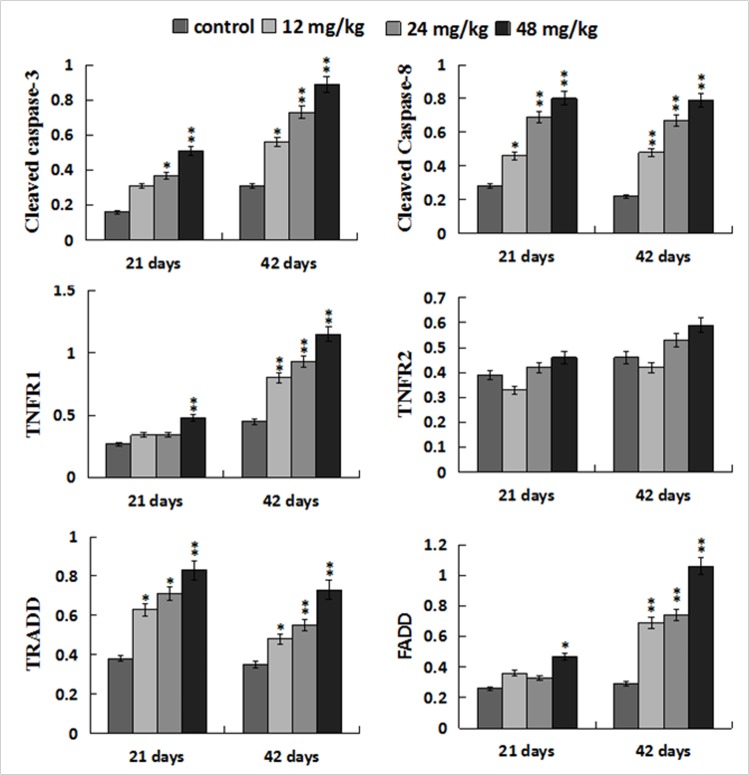
Changes of protein expression levels of apoptotic parameters associated to TNF-α signaling pathway in the liver Data are presented with the mean ± standard deviation (n=8). *p < 0.05, compared with the control group; **p < 0.01, compared with the control group.

The protein levels of cleaved caspase-8 and TRADD were significantly increased (*p* < 0.05 or *p* < 0.01) in the 12, 24, 48 mg/kg groups at 21 and 42 days of the experiment when compared with those in the control group. At the same time, the protein levels of TNF-R1 and FADD were higher (*p* < 0.05 or *p* < 0.01) in the 48 mg/kg group at 21 days of the experiment and in the 12, 24, 48 mg/kg groups at 42 days of the experiment than these in the control group. The cleaved caspase-3 protein levels were increased (*p* < 0.05 or *p* < 0.01) in the three NaF-treated groups at 21 and 42 days of the experiment except in the 12 mg/kg group at 21 days of the experiment. However, the TNF-R2 protein expression levels were not obviously changed. The results were shown in Figures 13 and 14.

## DISCUSSION

This study defines the NaF-induced hepatic oxidative stress and apoptosis as well as their relationship. Also, NaF-induced apoptosis through TNF-R1 signal pathway has been firstly reported in the liver at present.

It is well known that fluoride toxicity is associated with ROS induction [55]. In the present study, the results have proved that NaF can enhance hepatocellular ROS production levels in a time- and dose-dependent manner. Excessive ROS production can lead to lipid peroxidation [56], and MDA is the important indicator of lipid peroxidation [57, 58]. Our results in this study showed that MDA contents were increased in the liver of NaF-treated groups, which were consistent with the increased ROS production levels. The imbalance between ROS and the antioxidants can cause oxidative stress. The increased ROS levels may indicate the reduction of cellular antioxidant defenses. The current study showed that NaF decreased activities of antioxidant enzymes (SOD, CAT, GST and GSH-PX), and GSH contents in the liver, which were in line with our earlier studies on the effect of NaF on the antioxidant enzymes [12, 16, 27, 34]. SOD and CAT are important antioxidant enzymes, playing a major role in ROS elimination [59]. Non-enzymatic scavengers such as GSH are also involved in scavenging ROS, and the GSH dysfunction could aggravate the organ injury [60]. GSH-PX can promote the reaction between GSH and H_2_O_2_ in order to achieve the purpose of eliminating peroxide [61]. Therefore, the decreased GSH-PX activities caused by NaF in this study are closely correlated to the reduction of GSH contents. To show the molecular basis of the changes of antioxidant enzyme activities, the mRNA expression levels of CuZn-SOD, Mn-SOD, GST, CAT, GSH-Px were detected in the present study. The results showed that these antioxidant enzyme mRNA expression levels were increased in the NaF-treated groups, which were consistent with the reduction of their activities. The above results clearly indicated that NaF not only can promote the ROS production, but also inhibit the antioxidant enzyme mRNA expression in the liver. Then the imbalance between ROS and antioxidative function leads to the oxidative stress, which contributes to the occurrence of hepatocellular apoptosis.

It has been accepted that oxidative stress is an apoptotic inducer and some agents that induce apoptosis are either oxidants or stimulators of cellular oxidative metabolism. Conversely, many apoptosis inhibitors have antioxidative activities or can enhance the antioxidative defense ability [62, 63]. Recently, there are studies on revealing the pivotal role of ROS and oxidative stress in inducing apoptosis [64, 65]. In the present study, the detection of flow cytometry demonstrated that percentages of apoptotic hepatocytes were higher in the NaF-treated groups than those in the control group. In order to reveal the apoptosis mechanism induced by NaF, we further observed the expression of death receptor signal pathway involved in hepatocellular apoptosis. Guicciardi et al. [66] has found that death receptors are widely expressed in all liver cells. In TNF-receptor family, TNF-α has two receptors, TNF-R1 and TNF-R2. TNF-R1 play an important role in inducing apoptosis [67, 68]. However, TNF-R2 mainly activates the anti-apoptotic pathway [69]. Combination of the ligands and receptors recruits the adaptor proteins FADD and TNFR-associated death domain (TRADD), and then initiates the formation of death-inducing signaling complex (DICS), which further activates caspase-8, as a central mediator of death receptor signal pathway [70]. Activated caspase-8 catalyzes the caspase-3 proteolysis and drives the cascade reactions of downstream. In this study, we found that NaF treatment increased mRNA and protein expression levels of TNF-R1, FADD, TRADD, caspase-3 and caspase-8, which confirmed that TNF-R1 signaling pathway played a pivotal role in NaF-induced hepatocellular apoptosis.

Based on the above-mentioned discussion and the results in the present study, NaF-caused oxidative stress and apoptosis finally impaired hepatocytes and hepatic function, which was strongly supported by the histopathological lesions and increased serum AST, ALT, AKP activities and TBIL contents.

## CONCLUSIONS

The results show that NaF exposure induces hepatic oxidative stress and apoptosis. Oxidative stress is involved in the process of apoptotic occurrence, and can be triggered by promoting ROS production and reducing antioxidant function. NaF-caused oxidative stress and apoptosis finally impaired hepatocytes and hepatic function, which was strongly supported by the hepatocellular lesions histopathologically and increased serum AST, ALT, AKP activities and TBIL contents. Also, it has been demonstrated that NaF induces hepatocellular apoptosis through TNF-R1 signal pathway in mice.

## MATERIALS AND METHODS

### Chemicals

Sodium fluoride was purchased from Chengdu Kelong Chemical Co., Ltd. (Chengdu, China). ROS Assay kit (S0033) was obtained from Beyotime Biotechnology, China. Reagent kits for determination of biochemical parameters were purchased from Nanjing Jiancheng Bioengineering Institute of China (Nanjing, China). RNAiso Plus, Prim-ScriptTM RT reagent Kit and SYBR® Premix Ex TaqTM II were purchased from Takara Biotechnology (Dalian) Co., Ltd. (Dalian, Liaoning, China). All other chemicals used in the experiment were analytical grade.

### Animals and treatment

240 ICR mice were provided by Chengdu Dossy Experimental Animals Co., Ltd. [License No. SCXK (Sichuan) 2008-24] and were randomly divided into 4 equal groups, and housed in separate cages. The control group received distilled water. The low-, medium-, and high-fluoride groups were oral administered with NaF at a dose of 12, 24 and 48 mg/kg body weight for consecutively 42 days, and the gavage volume was 1ml/100g body weight respectively. All of the mice had free access to food and water.

All experimental procedures involving the use of mice were approved by the Animal Care and Use Committee, Sichuan Agricultural University.

### Histopathological observation

At 21 and 42 days of the experiment, eight mice in each group were humanely killed, and the livers were immediately taken out and fixed in 4% buffered formaldehyde, dehydrated through graded alcohol, and routinely processed in paraffin. Thin slices (5μm) of each tissue were sliced through routine microtomy. Slices were stained with hematoxylin and eosin (H&E) and then were examined under optical microscope.

### Detection of hepatic functional parameters

At 21 and 42 days of the experiment, blood was taken from retro-ocular artery of eight mice in each group without anticoagulant. Serum samples were collected after centrifuged at 3000 rpm for 15 min. Serum ALT, AST, AKP activities and TBIL contents were measured according to the directions of biological reagent kits purchased from Nanjing Jiancheng Bioengineering Institute of China (Nanjing, China).

### Detection of oxidative stress parameters in the liver

At 21 and 42 days of the experiment, after eight mice in each group were sacrificed, the livers were immediately removed, and washed using chilled saline solution, weighed, homogenized in nine volumes of ice-cold 0.9% NaCl solution and centrifuged at 3500 rpm for 10 min at 4°C. The supernatants were collected for detecting the activities of CAT, SOD, GST and GSH-Px, and contents of GSH and MDA by biochemical methods following the instructions of the corresponding reagent experiment kits (CAT, A007-1; SOD, A001-1; GST, A004; GSH-Px, A005; GSH, A006-2; MDA, A003-1; total protein, A045-3, purchased from Nanjing Jiancheng Bioengineering Institute of Nanjing, China) after determining the total protein contents in the supernatant by the Bradford method [71].

### Detection of antioxidant enzyme and apoptosis parameter mRNA expression by qRT-PCR

At 21 and 42 days of the experiment, livers from eight mice in each group were respectively stored in liquid nitrogen, and homogenized with liquid nitrogen for RNA extraction. The methods of RNA extraction were same as the described by Yin al. [72]. The total RNA of the liver were extracted using RNAiso Plus (9109; Takara, China). The cDNA was synthesized using a Prim-Script™ RT reagent Kit (RR047A, Takara, China) following the manufacture’s instruction. The cDNA sequences of CuZn-SOD, Mn-SOD, GST, CAT, GSH-Px and cleaved caspase-3, cleaved caspase-8, TNF-R1, TNF-R2, FADD, TRADD were referred from NCBI, and β-actin was used as reference gene. The primers were designed and synthesized by Sangon Bioteech Biological Technology Company (Shanghai, China) (Table 1).

**Table 1:** The list of oligonucleotides used as primers in qRT-PCR analysis of gene expression

Primer name	Primer sequence (5′-3′)				
	Forward primer	Reverse primer	Accession number	product size	Tm (°C)
CAT	CCTATTGCCGTTCGATTCTC	CCCACAAGATCCCAGTTACC	NM-009804	119bp	61
Mn-SOD	AACTCAGGTCGCTCTTCAGC	CTCCAGCAACTCTCCTTTGG	NM-013671	113bp	61
CuZn-SOD	GGGTTCCACGTCCATCAGTA	CAGGTCTCCAACATGCCTCT	NM-011434	113bp	61
GSH-Px	CCAGGAGAATGGCAAGAATG	AAGGTAAAGAGCGGGTGAGC	NM-008160	102bp	57
GST	GGGATTGGCTGTGATGAGAT	AGGTAGGATGAATGGCAACTG	NM-019946	121bp	61
Caspase-3	TCTGACTGGAAAGCCGAAAC	GCAAGCCATCTCCTCATCA	NM-009810	103bp	57
Caspase-8	GCTGCCCTCAAGTTCCTGT	GATTGCCTTCCTCCAACATC	NM-009812	118bp	61
TNF-R1	AATGCAGACCTTGCGATTCT	CATCTCCAGCCTCTCGATCT	NM-011609	114bp	59
TNF-R2	CATCACTGGGTCTGCTGATG	TCCTGGGATTTCTCATCAGG	NM-011610	124bp	57
FADD	CGTGAGAAACGAAAGCTGG	CTGCAGTAGATCGTGTCGGC	NM-010175	142bp	60
TRADD	TGGCTGACTGATGAAGAGCG	CACACGTCAGTTTGCAGAGC	NM-001033161	112bp	60
β-actin	GCTGTGCTATGTTGCTCTAG	CGCTCGTTGCCAATAGTG	NM-007393	117bp	59

qRT-PCR reactions was performed on a Thermal Cycler (C1000, BIO RAD, USA) Using SYBR® Premix Ex Taq^TM^II (RR820A, Takara, China). Gene expression values of the control group at 21 and 42 days of the experiment were used for gene expression calibration. The results were analyzed with 2^−ΔΔCT^ method [73].

### Detection of apoptosis parameter protein expression by western blot

Proteins were extracted with RIPA lysis buffer and the protein concentration was quantitated by BCA protein assay reagent. Protein samples were separated by (10%-15% gels) SDS-PAGE and transferred to nitrocellulose filter membranes. The membranes were blocked in 5% skim milk for 1h and incubated with the primary antibodies overnight at 4°C. The primary antibodies were cleaved caspase-3, cleaved caspase-8, TNF-R1, TNF-R2, FADD, TRADD. The membranes were then washed with PBST (PBS-Tween). Blots were visualized by ECL^TM^ (BIO-RAD) and X-ray film. The statistical data of protein expression was done with imageJ2x software.

### Detection of apoptosis by flow cytometry

At 21 and 42 days of the experiment, after eight mice in each group were sacrificed, livers were immediately moved and ground to form a cell suspension which could be filtered through the 300-mesh nylon. The cells were washed twice with cold PBS (phosphate buffer solution pH 7.2-7.4) and suspended in PBS at a concentration of 1×10^6^ cells/mL, and 100μL cell suspension was transferred into a 5mL tubes, and stained with PE Annexin V and 7-aminoactinomycin (7-AAD). The mixture was gently vibrated and incubated for 15min in the dark place, and then 400 μL of 1× binding buffer was added to each tube. Finally, the hepatic apoptosis was analyzed by FACSCalibur (BD FACS Calibur).

### Detection of hepatocellular ROS production by flow cytometry

At 21 and 42 days of the experiment, after eight mice in each group were sacrificed, livers were taken to measure the levels of ROS production by flow cytometry. Livers were crushed, filtered with 350 mesh nylon membrane, centrifuged (600 × g, 5min), and adjusted to a cell density of 1.0×10^6^ cells/ml with phosphate-buffered saline (PBS). 300 μL cell suspensions were taken and transferred to another centrifuge tube, and stained with 10μM DCFH-DA for 20 min at 37°C. Then the cells were washed with PBS and centrifuged (600 × g, 5min) once more. The supernatant was discarded, and cells were resuspended in 0.5 ml PBS and counted by BD FACS Calibur flow cytometer within 45 min.

### Statistical analysis

The significance of difference was analyzed by the SPSS version 17.0. The results were shown as means ± standard deviation. The analysis was performed with the one-way analysis of variance (ANOVA). The differences between control and experimental group(s) at *p* < 0.05 or *p* < 0.01 were considered significant.

## References

[1] Jha SK, Mishra VK, Sharma DK, Damodaran T (2011). Fluoride in the environment and its metabolism in humans. Rev Environ Contam Toxicol.

[2] Song G, Wang RL, Chen ZY, Zhang B, Wang HL, Liu ML, Gao JP, Yan XY (2014). Toxic effects of sodium fluoride on cell proliferation and apoptosis of Leydig cells from young mice. J Physiol Biochem.

[3] Meenakshi, Maheshwari RC (2006). Fluoride in drinking water and its removal. J Hazard Mater.

[4] Farooqi A, Masuda H, Firdous N (2007). Toxic fluoride and arsenic contaminated groundwater in the Lahore and Kasur districts, Punjab, Pakistan and possible contaminant sources. Environ Pollut.

[5] Ozsvath D (2009). Fluoride and environmental health: a review. Rev Env Iron Sci Bio.

[6] Shivarajashankara YM, Rao SM, Rao SH, Shivashankara AR, Bhat PG (2002). Histological changes in the brain of young fluoride-intoxicated rats. Fluoride.

[7] Chen T, Cui Y, Bai C, Gong T, Peng X, Cui H (2009). Decreased percentages of the peripheral blood T-cell subsets and the serum IL-2 contents in chickens fed on diets excess in fluorine. Biol Trace Elem Res.

[8] Deng YB, Cui H, Peng X, Fang J, Zuo Z, Deng J, Luo Q (2014). Effects of high dietary fluoride on serum biochemistry and oxidative stress parameters in broiler chickens. Health.

[9] Deng Y, Cui H, Peng X, Fang J, Zuo Z, Deng J, Luo Q (2013). Effects of high dietary fluorine on erythrocytes and erythrocyte immune adherence function in broiler chickens. Biol Trace Elem Res.

[10] Deng YB, Cui HM, Peng X, Fang J, Zuo ZC, Deng JL, Luo Q (2013). High dietary fluorine alteration of serum cytokine and immunoglobulin in broilers. Fluoride.

[11] Gong T, Chen T, Bai CM, Peng X, Cui HM (2009). Effect of dietary high fluorine on the cell cycle and apoptosis of liver in chickens. Chinese J Anim Vet Sci.

[12] Gong T, Bai CM, Chen T, Peng X, Cui HM (2009). Effect of high fluorine on the antioxidant function and ultrastructure of liver in chickens. Chinese J Anim Vet Sci.

[13] Bai C, Chen T, Cui Y, Gong T, Peng X, Cui HM (2010). Effect of high fluorine on the cell cycle and apoptosis of renal cells in chickens. Biol Trace Elem Res.

[14] Bai CM, Peng X, Gong T, Chen T, Cui HM (2010). Effect of high fluorine on the antioxygen function of kidney in chickens. Chinese J Anim Vet Sci.

[15] Bai CM, Chen T, Gong T, Peng X, Cui HM (2010). Pathological effect of high fluorine on kidney and the related biochemical parameters of serum in the chicken. Chinese J Anim Vet Sci.

[16] Chen T, Cui H, Cui Y, Bai C, Gong T, Peng X (2011). Cell-cycle blockage associated with increased apoptotic cells in the thymus of chickens fed on diets high in fluorine. Hum Exp Toxicol.

[17] Chen T, Cui H, Cui Y, Bai C, Gong T (2011). Decreased antioxidase activities and oxidative stress in the spleen of chickens fed on high-fluorine diets. Hum Exp Toxicol.

[18] Chen T, Cui Y, Bai CM, Gong T, Peng X, Cui HM (2009). Increased apoptotic lymphocyte population in the spleen of young chickens fed diets high in fluorine. Fluoride.

[19] Chen T, Cui Y, Gong T, Bai CM, Peng X, Cui HM (2009). Inhibition of splenocyte proliferation and spleen growth in young chickens fed high fluoride diets. Fluoride.

[20] Chen T, Gong T, Bai CM, Peng X, Cui HM (2009). Effect of dietary high fluorine on the morphologic structure, cell cycle and apoptosis of bursa of Fabricius in broilers. Chinese J Anim Vet Sci.

[21] Liu J, Cui HM, Peng X, Fang J, Zuo ZC, Wang HS, Wu BY, Deng YB, Wang KP (2012). Changes induced by high dietary fluorine in the cecal tonsil cytokine content of broilers. Fluoride.

[22] Liu J, Cui H, Peng X, Fang J, Zuo Z, Deng J, Wang H, Wu B, Deng Y, Wang K (2013). Decreased IgA^+^ B cells population and IgA, IgG, IgM contents of the cecal tonsil induced by dietary high fluorine in broilers. Int J Environ Res Public Health.

[23] Liu J, Cui HM, Peng X, Fang J, Zuo ZC, Wang HS, Wu BY, Deng YB, Wang KP (2012). Decreased percentages of T-cell subsets and IL-2 contents in the cecal tonsil of broilers fed diets high in fluorine. Fluoride.

[24] Liu J, Cui H, Peng X, Fang J, Zuo Z, Wang H, Wu B, Deng Y, Wang K (2013). Dietary high fluorine induces apoptosis and alters Bcl-2, Bax, and caspase-3 protein expression in the cecal tonsil lymphocytes of broilers. Biol Trace Elem Res.

[25] Liu J, Cui HM, Peng X, Fang J, Zuo ZC, Wang HS, Wu BY, Deng YB, Wang KP (2012). High dietary fluorine induction of oxidative damage in the cecal tonsil of broilers. Fluoride.

[26] Luo Q, Cui H, Peng X, Fang J, Zuo Z, Deng J, Liu J, Deng Y (2016). Dietary high fluorine alters intestinal microbiota in broiler chickens. Biol Trace Elem Res.

[27] Luo Q, Cui H, Peng X, Fang J, Zuo Z, Deng J, Liu J, Deng Y (2013). Intestinal IgA^+^ cell numbers as well as IgA, IgG, and IgM contents correlate with mucosal humoral immunity of broilers during supplementation with high fluorine in the diets. Biol Trace Elem Res.

[28] Luo Q, Cui HM, Peng X, Fang J, Zuo ZC, Liu J, Wu BY, Wang HS, Deng YB, Huang JY (2012). Intestinal oxidative stress in broilers caused by high dietary fluorine. Fluoride.

[29] Luo Q, Cui H, Peng X, Fang J, Zuo Z, Deng J, Liu J, Deng Y (2013). Suppressive effects of dietary high fluorine on the intestinal development in broilers. Biol Trace Elem Res.

[30] Luo Q, Cui H, Peng X, Fang J, Zuo Z, Liu J, Wu B, Deng Y (2013). The association between cytokines and intestinal mucosal immunity among broilers fed on diets supplemented with fluorine. Biol Trace Elem Res.

[31] Kuang P, Deng H, Cui H, Chen L, Guo H, Fang J, Zuo Z, Deng J, Wang X, Zhao L (2016). Suppressive effects of sodium fluoride on cultured splenic lymphocyte proliferation in mice. Oncotarget.

[32] Deng H, Kuang P, Cui H, Chen L, Fang J, Zuo Z, Deng J, Wang X, Zhao L, Zhao L (2016). Sodium fluoride induces apoptosis in cultured splenic lymphocytes from mice. Oncotarget.

[33] Deng H, Kuang P, Cui H, Chen L, Luo Q, Fang J, Zuo Z, Deng J, Wang X, Zhao L (2016). Sodium fluoride (NaF) induces the splenic apoptosis via endoplasmic reticulum (ER) stress pathway *in vivo* and *in vitro.*. Aging (Albany NY).

[34] Kuang P, Deng H, Cui H, Chen L, Fang J, Zuo Z, Deng J, Wang X, Zhao L (2017). Sodium fluoride (NaF) causes toxic effects on splenic development in mice. Oncotarget.

[35] Luo Q, Cui H, Deng H, Kuang P, Liu H, Lu Y, Fang J, Zuo Z, Deng J, Li Y, Wang X, Zhao L (2017). Histopathological findings of renal tissue induced by oxidative stress due to different concentrations of fluoride. Oncotarget.

[36] Cabiscol E, Tamarit J, Ros J (2000). Oxidative stress in bacteria and protein damage by reactive oxygen species. Int Microbiol.

[37] Mittler R (2002). Oxidative stress, antioxidants and stress tolerance. Trends Plant Sci.

[38] Kale M, Rathore N, John S, Bhatnagar D (1999). Lipid peroxidative damage on pyrethroid exposure and alterations in antioxidant status in rat erythrocytes: a possible involvement of reactive oxygen species. Toxicol Lett.

[39] Shivarajashankara YM, Shivashankara AR, Bhat PG, Rao SH (2003). Lipid peroxidation and antioxidant systems in the blood of young rats subjected to chronic fluoride toxicity. Indian J Exp Biol.

[40] Guo XY (2003). Oxidative stress from fluoride-induced hepatotoxicity in rats. Fluoride.

[41] Ranjan R, Swarup D, Patra R, Izatnagar C (2009). Oxidative stress indices in erythrocytes, liver, and kidneys of fluoride-exposed rabbits. Fluoride.

[42] Ghosh D, Das Sarkar S, Maiti R, Jana D, Das UB (2002). Testicular toxicity in sodium fluoride treated rats: association with oxidative stress. Reprod Toxicol.

[43] Bharti VK, Srivastava RS (2009). Fluoride-induced oxidative stress in rat’s brain and its amelioration by buffalo (Bubalus bubalis) pineal proteins and melatonin. Biol Trace Elem Res.

[44] Nabavi SF, Nabavi SM, Ebrahimzadeh MA, Sh E (2011). The protective effect of curcumin against sodium fluoride-induced oxidative stress in rat heart. Arch Biol Sci.

[45] Mukhopadhyay D, Chattopadhyay A (2014). Induction of oxidative stress and related transcriptional effects of sodium fluoride in female zebrafish liver. Bull Environ Contam Toxicol.

[46] Zhou BH, Zhao J, Liu J, Zhang JL, Li J, Wang HW (2015). Fluoride-induced oxidative stress is involved in the morphological damage and dysfunction of liver in female mice. Chemosphere.

[47] Zhan XA, Wang M, Xu ZR, Li WF, Li JX (2006). Evaluation of caspase-dependent apoptosis during fluoride-induced liver lesion in pigs. Arch Toxicol.

[48] Song GH, Gao JP, Wang CF, Chen CY, Yan XY, Guo M, Wang Y, Huang FB (2014). Sodium fluoride induces apoptosis in the kidney of rats through caspase-mediated pathways and DNA damage. J Physiol Biochem.

[49] Liu L, Zhang Y, Gu H, Zhang K, Ma L (2015). Fluorosis induces endoplasmic reticulum stress and apoptosis in osteoblasts in vivo. Biol Trace Elem Res.

[50] Song GH, Huang FB, Gao JP, Liu ML, Pang WB, Li W, Yan XY, Huo MJ, Yang X (2015). Effects of Fluoride on DNA Damage and Caspase-Mediated Apoptosis in the Liver of Rats. Biol Trace Elem Res.

[51] Zhan XA, Wang M, Xu ZR, Li WF, Li JX (2006). Evaluation of caspase-dependent apoptosis during fluoride-induced liver lesion in pigs. Arch Toxicol.

[52] Cao J, Chen J, Wang J, Jia R, Xue W, Luo Y, Gan X (2013). Effects of fluoride on liver apoptosis and Bcl-2, Bax protein expression in freshwater teleost, Cyprinus carpio. Chemosphere.

[53] Danial NN, Korsmeyer SJ (2004). Cell death: critical control points. Cell.

[54] Galluzzi L, Vitale I, Abrams JM, Alnemri ES, Baehrecke EH, Blagosklonny MV, Dawson TM, Dawson VL, El-Deiry WS, Fulda S, Gottlieb E, Green DR, Hengartner MO (2012). Molecular definitions of cell death subroutines: recommendations of the Nomenclature Committee on Cell Death 2012. Cell Death Differ.

[55] Apel K, Hirt H (2004). Reactive oxygen species: metabolism, oxidative stress, and signal transduction. Annu Rev Plant Biol.

[56] Farmer EE, Mueller MJ (2013). ROS-mediated lipid peroxidation and RES-activated signaling. Annu Rev Plant Biol.

[57] Draper HH, Hadley M (1990). Malondialdehyde determination as index of lipid peroxidation. Methods Enzymol.

[58] Nabavi SF, Nabavi SM, Mirzaei M, Moghaddam AH (2012). Protective effect of quercetin against sodium fluoride induced oxidative stress in rat’s heart. Food Funct.

[59] Thangapandiyan S, Miltonprabu S (2013). Epigallocatechin gallate effectively ameliorates fluoride-induced oxidative stress and DNA damage in the liver of rats. Can J Physiol Pharmacol.

[60] Liang Q, Sheng Y, Jiang P, Ji L, Xia Y, Min Y, Wang Z (2011). The gender-dependent difference of liver GSH antioxidant system in mice and its influence on isoline-induced liver injury. Toxicology.

[61] Chinoy N, Sharma A, Patel T, Memon R, Dd J, Ahmedabad (2004). Recovery from fluoride and aluminium induced free radical liver toxicity in mice. Fluoride.

[62] Chandra J, Samali A, Orrenius S (2000). Triggering and modulation of apoptosis by oxidative stress. Free Radic Biol Med.

[63] Ratan RR, Murphy TH, Baraban JM (1994). Oxidative stress induces apoptosis in embryonic cortical neurons. J Neurochem.

[64] Kannan K, Jain SK (2000). Oxidative stress and apoptosis. Pathophysiology.

[65] Harsdorf RV (2007). Reactive oxygen species and apoptosis. Apoptosis Card Bio.

[66] Guicciardi ME, Gores GJ (2005). Apoptosis: a mechanism of acute and chronic liver injury. Gut.

[67] Schulze-Osthoff K, Ferrari D, Los M, Wesselborg S, Peter ME (1998). Apoptosis signaling by death receptors. Eur J Biochem.

[68] Ashkenazi A, Dixit VM (1998). Death receptors: signaling and modulation. Science.

[69] Fotin-Mleczek M, Henkler F, Samel D, Reichwein M, Hausser A, Parmryd I, Scheurich P, Schmid JA, Wajant H (2002). Apoptotic crosstalk of TNF receptors: TNF-R2-induces depletion of TRAF2 and IAP proteins and accelerates TNF-R1-dependent activation of caspase-8. J Cell Sci.

[70] Thorburn A (2004). Death receptor-induced cell killing. Cell Signal.

[71] Arruda P, Sodek L, da Silva WJ (1982). Lysine-ketoglutarate reductase activity in developing maize endosperm. Plant Physiol.

[72] Yin S, Guo H, Cui H, Peng X, Fang J, Zuo Z, Deng J, Wang X, Tang K, Li J (2016). Nickel chloride (NiCl_2_) induces histopathological lesions via oxidative damage in the broiler’s Bursa of Fabricius. Biol Trace Elem Res.

[73] Livak KJ, Schmittgen TD (2001). Analysis of relative gene expression data using real-time quantitative PCR and the 2(-Delta Delta C(T)) Method. Methods.

